# Clinico‑Radiologic Probable Acute Haemorrhagic Leukoencephalitis Triggered by Legionella Pneumonia

**DOI:** 10.7759/cureus.98617

**Published:** 2025-12-07

**Authors:** Eslam Abdelaziz

**Affiliations:** 1 Acute Medicine, William Harvey Hospital, East Kent Hospitals University NHS Foundation Trust, Ashford, GBR

**Keywords:** acute disseminated encephalomyelitis, acute disseminated encephalomyelitis (adem), acute haemorrhagic leukoencephalopathy, acute hemorrhagic leukoencephalitis (ahle), legionella

## Abstract

Acute haemorrhagic leukoencephalitis (AHLE) is a rare disease characterised by focal neurological symptoms, commonly following a fulminant course and progressing to coma or, in some cases, death. Although the exact pathophysiology is unclear, AHLE commonly follows infections and is thought to be a post-infectious autoimmune demyelinating neuropathy. Therefore, the mainstay of treatment is immunosuppression.

We report a rare case of a 66-year-old male smoker with severe Legionella pneumonia requiring intubation and ventilation, who subsequently developed a reduced Glasgow Coma Scale (GCS) and generalised weakness. Magnetic resonance imaging (MRI) demonstrated extensive symmetrical microhaemorrhages consistent with AHLE. Based on clinical and radiological findings, he was treated with intravenous steroids and plasma exchange, ultimately making a full recovery with normal GCS and normal limb power.

## Introduction

Acute haemorrhagic leukoencephalitis (AHLE), also known as Weston-Hurst syndrome, first described in 1941, is a life‑threatening condition presenting with a wide range of neurological disturbances including visual field defects, gaze abnormalities, pseudobulbar palsy, aphasia, mutism, incontinence, and motor and sensory deficits [1]. Additional symptoms may include photophobia, meningeal signs, confusion, lethargy, and progression to coma. Mortality reaches 46.5%, with 39.5% of survivors left with neurological impairment, and only 14% achieving full recovery according to a 2020 systematic review [2]. 

AHLE remains extremely rare. A 2020 systematic review identified only 43 adult cases over the preceding 20 years, with 67% occurring in males [2]. Of these, 35% were associated with infectious pathogens, 19% with upper respiratory tract infections without identifiable pathogens, and 12% with autoimmune disease. Only one published case links AHLE with Legionella pneumonia [3]. 

Although its exact pathogenesis remains unclear, AHLE is believed to represent an infection‑triggered autoimmune inflammatory response involving cross‑reactivity between microbial antigens and myelin components, causing demyelination and tissue damage [4]. AHLE shares several clinical and pathological features with acute demyelinating encephalomyelitis (ADEM), a less severe demyelinating condition more common in children [1,2]. The main radiological difference between AHLE and ADEM is that the inflammatory lesions in the former are associated with haemorrhagic necrosis and extensive white matter oedematous changes, unlike ADEM [5]. Treatments typically include high‑dose glucocorticoids, plasma exchange, and intravenous immunoglobulin [2]. 

## Case presentation

A 66-year-old male smoker with no significant past medical history presented to the emergency department after being found collapsed at home three days following return from a holiday in Europe. He had a five-day history of fever, lethargy, coryza symptoms, and a non‑productive cough. On arrival, his temperature was 38.1°C, blood pressure 104/63 mmHg, GCS was 15 and oxygen saturation was 80% on room air, which improved to 92% on 15 L/min oxygen. He had bilateral mid‑zone crackles with no focal neurological deficits and was alert and oriented.

**Figure 1:**
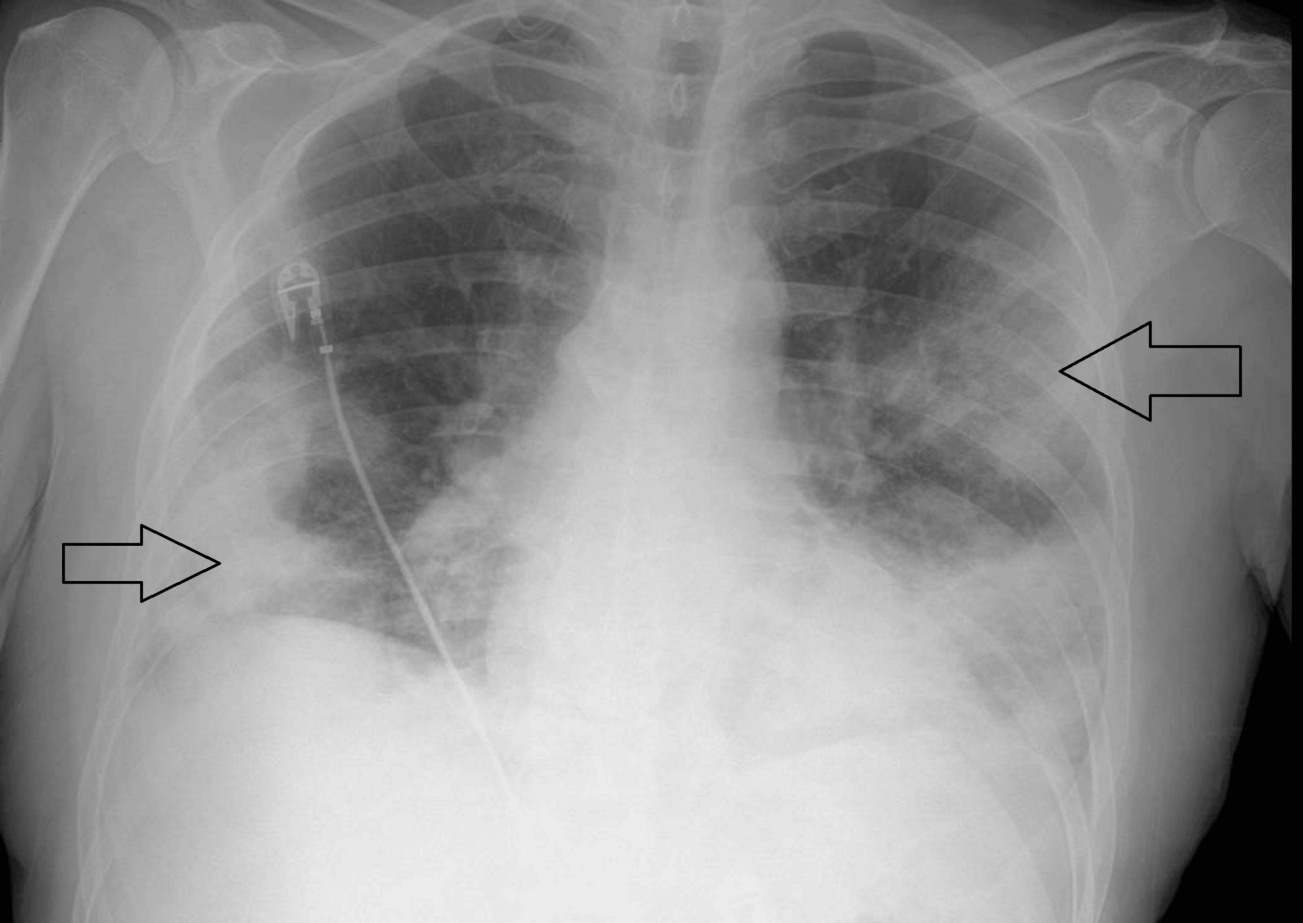
Chest X-ray showing bilateral patchy consolidation.

Chest X‑ray demonstrated bilateral basal consolidation (Figure 1). Blood tests were done, showing a significantly raised C-reactive protein (CRP), and an arterial blood gas on room air confirmed type one respiratory failure. The blood test and arterial blood gas results can be seen in Table 1. COVID‑19 and influenza tests were negative. Legionella urinary antigen was positive, and appropriate antibiotics were commenced.

**Table 1:** Blood test results on admission and arterial blood gas on room air. g/L: grams per liter; L: liter; mg/L: milligrams per liter; mmol/L: millimoles per liter; PT: prothrombin time; APTT: activated partial thromboplastin time; mg/dL: milligrams per deciliter; PCO2: partial pressure of carbon dioxide; kPa: Kilopascals; PO2: partial pressure of oxygen; HCO3: bicarbonate; SaO2: arterial oxygen saturation; O2Hb: oxyhaemoglobin.

Parameters	Patient values	Reference range
Admission blood tests:		
Haemoglobin	129 g/L	130 – 180 g/L
Platelets	264 x10^9/L	140 – 400 x10^9/L
White cell count	11.4 x10^9/L	3.6 – 11 x10^9/L
Neutrophils	10.8 x10^9/L	1.8 – 7.5 x10^9/L
C-reactive protein (CRP)	547 mg/L	Less than 5 mg/L
Sodium	132.2 mmol/L	136 – 145 mmol/L
PT	10.3 seconds	9 – 13 seconds
APTT	19.2 seconds	22 – 36 seconds
Fibrinogen	> 4.5 mg/dL	1.5 – 4.5 mg/dL
Arterial blood gas on room air:		
pH	7.433	7.350 – 7.450
PCO2	4.11 kPa	4.27 – 6.00 kPa
PO2	5.49 kPa	11.07 – 14.40 kPa
HCO3	20.2 mmol/L	22.00 – 26.00 mmol/L
SaO2	82%	94 – 98%
O2Hb	80.2%	94 – 98%

**Figure 2:**
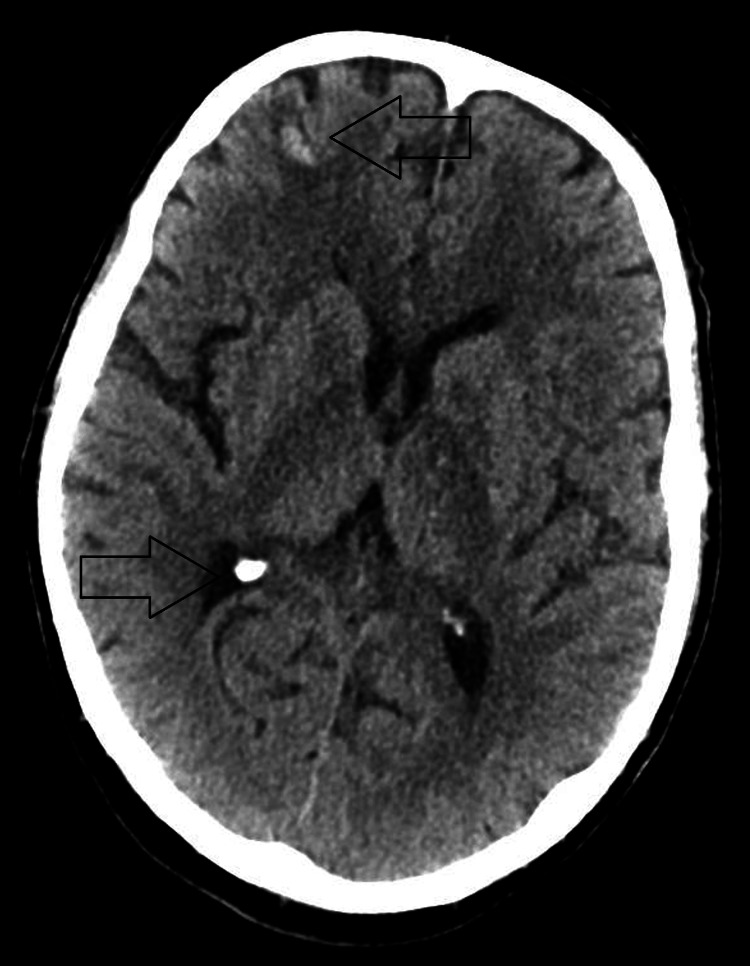
CT head showing hyperdensity lesions suspicious for microhaemorrhages involving the right frontal subcortical grey matter and basal ganglia.

**Figure 3:**
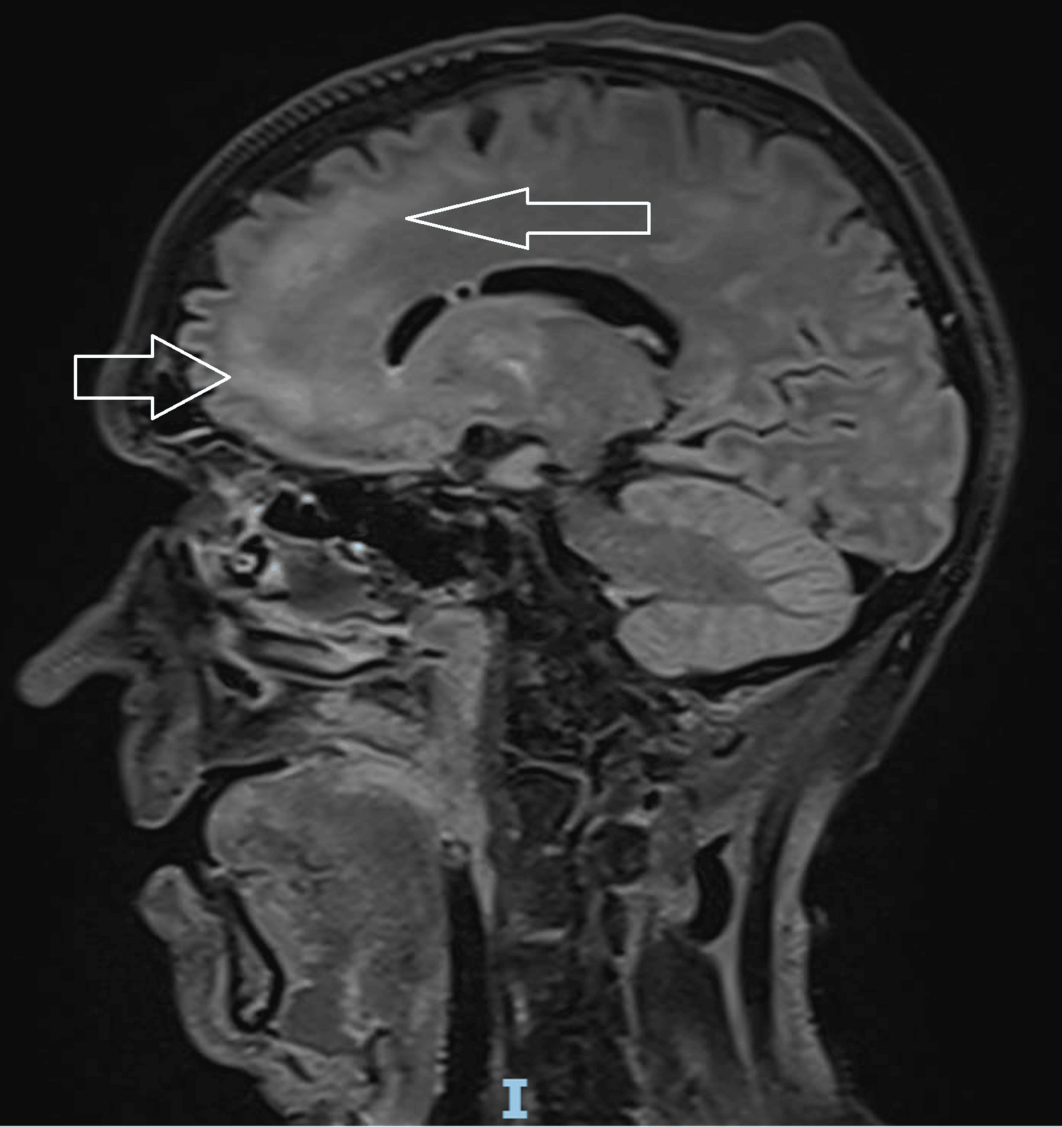
MRI head T2 sagittal view showing T2 hyperintensity throughout the supratentorial white matter.

**Figure 4:**
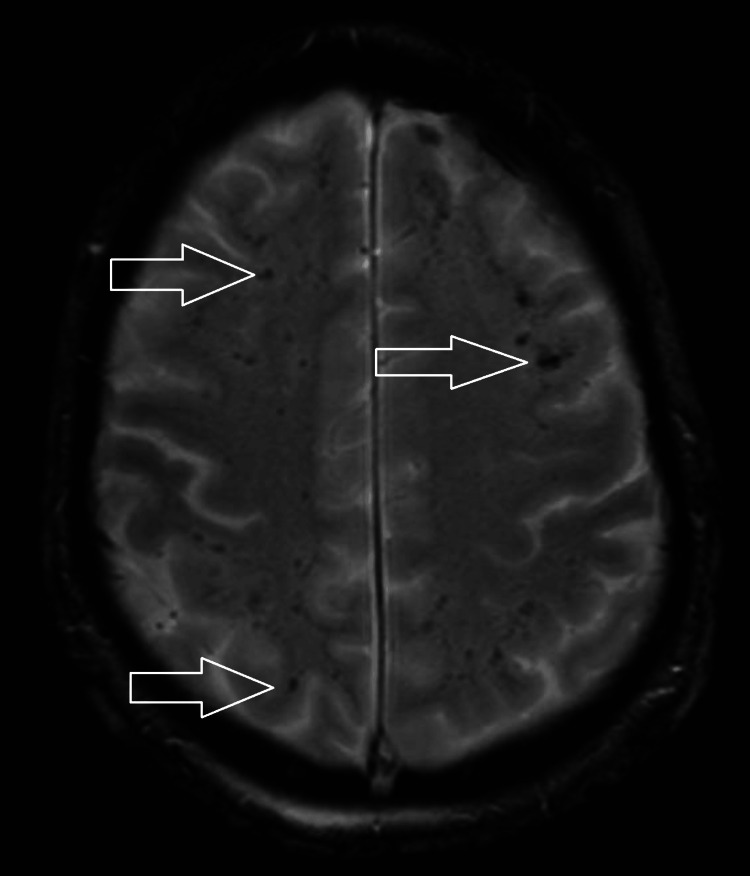
MRI head axial view. Image from the susceptibility-weighted sequence showing innumerable foci of increased susceptibility indicating microhaemorrhages.

He was admitted to the Intensive Care Unit (ICU) on the day of presentation to the hospital for escalating oxygen requirements and required intubation, sedation, and mechanical ventilation for a week post admission. Sedation was discontinued after one week, but two days after stopping all sedative medications, he remained unresponsive with a GCS of 3/15, no spontaneous limb movement and normal direct and consensual pupillary reflexes. He also had normal, bilaterally symmetrical deep tendon reflexes in upper and lower limbs with equivocal plantar reflexes bilaterally. The severity of the patient's condition was suspicious for a central nervous system (CNS) disease and CT head 10 days into hospitalisation revealed multiple small supratentorial hyperdensities (Figure 2). MRI two days after showed extensive symmetrical T2 hyperintensities in the supratentorial white matter, predominantly in subcortical regions and internal capsules, suggestive of disseminated encephalomyelitis (Figure 3). Susceptibility-weighted imaging revealed innumerable foci of microhaemorrhage at the grey-white matter interface, typical for AHLE (Figure 4). The patient's neurological condition and GCS remained unchanged to this point.

Given the severity of the patient's neurological condition, and the poor outcomes of AHLE, neurologists advised treatment with intravenous methylprednisolone 1 g daily for three days, followed by five plasma exchange sessions and an oral prednisolone taper for AHLE based on the MRI findings and clinical presentation. Immediately after intravenous steroid therapy, his GCS improved to 11 (with incomplete documentation of component scores), and within one week, it increased to 15. Three weeks later, he regained limb power graded 3/5 (Medical Research Council scale) in all extremities. One month later, he was discharged with normal motor power and referred for community rehabilitation. At one‑year follow‑up, he remained clinically well with no relapse.

We considered critical illness‑associated cerebral microbleeds as a differential diagnosis, given the prolonged mechanical ventilation and hypoxaemia [6]. While extensive microhaemorrhages, diffusely involving the juxtacortical white matter and corpus callosum on MRI, can be seen in this disease, the extent of confluent T2 hyperintensities favoured an inflammatory demyelinating process consistent with AHLE rather than a pure microbleed syndrome. The robust response to steroids and plasma exchange also supports the diagnosis. While there are reports of multiple cerebral microbleeds associated with disseminated intravascular coagulation (DIC), possibly related to critical illness‑associated cerebral microbleeds [7], the coagulation profile was unremarkable, making DIC unlikely. Additionally, Myelin oligodendrocyte glycoprotein (MOG) antibodies were negative, making MOG antibody-associated disease (MOGAD) unlikely. This was tested due to the suggested potential association between AHLE and MOGAD [8].

## Discussion

We describe a rare case of AHLE associated with Legionella pneumonia, based on clinical and radiological diagnosis, with only one previously published case reporting the same association [3]. This case is notable for the patient’s complete neurological recovery following treatment with high-dose corticosteroids and plasma exchange, an outcome achieved in only 14% of cases in a recent systematic review [2].

Although MRI findings were strongly suggestive of AHLE, limitations of this case include the absence of cerebrospinal fluid analysis, electroencephalogram (EEG) or brain biopsy, which may aid diagnostic certainty. Nevertheless, the clinical picture of rapid neurological decline following respiratory infection, combined with the characteristic MRI abnormalities and marked improvement with immunosuppressive therapy, supports the diagnosis.

## Conclusions

AHLE is a rare, severe, and likely under-recognised condition due to limited awareness and the challenges of diagnosis. While MRI is essential for diagnosis, limitations to obtaining a confirmed diagnosis include the availability of certain investigations including cerebrospinal fluid analysis, EEG and brain biopsy.

With a significant mortality rate, early recognition is crucial. Clinicians should consider AHLE in patients presenting with acute neurological deterioration, particularly in the context of recent infection. Prompt immunosuppressive treatment, including high-dose corticosteroids, plasma exchange, and intravenous immunoglobulin, may improve outcomes.
